# Integrated analysis of potential gene crosstalk between non-alcoholic fatty liver disease and diabetic nephropathy

**DOI:** 10.3389/fendo.2022.1032814

**Published:** 2022-10-25

**Authors:** Qianqian Yan, Zihao Zhao, Dongwei Liu, Jia Li, Shaokang Pan, Jiayu Duan, Jiancheng Dong, Zhangsuo Liu

**Affiliations:** ^1^ Department of Integrated Traditional and Western Nephrology, The First Affiliated Hospital of Zhengzhou University, Zhengzhou, China; ^2^ Research Institute of Nephrology, Zhengzhou University, Zhengzhou, China; ^3^ Henan Province Research Center for Kidney Disease, Zhengzhou, China; ^4^ Key Laboratory of Precision Diagnosis and Treatment for Chronic Kidney Disease in Henan Province, Zhengzhou, China

**Keywords:** non-alcoholic fatty liver disease, diabetic nephropathy, crosstalk, LPL, SPP1, bioinformatics

## Abstract

**Background:**

Growing evidence indicates that non-alcoholic fatty liver disease (NAFLD) is related to the occurrence and development of diabetic nephropathy (DN). This bioinformatics study aimed to explore optimal crosstalk genes and related pathways between NAFLD and DN.

**Methods:**

Gene expression profiles were downloaded from Gene Expression Omnibus. CIBERSORT algorithm was employed to analyze the similarity of infiltrating immunocytes between the two diseases. Protein–protein interaction (PPI) co-expression network and functional enrichment analysis were conducted based on the identification of common differentially expressed genes (DEGs). Least absolute shrinkage and selection operator (LASSO) regression and Boruta algorithm were implemented to initially screen crosstalk genes. Machine learning models, including support vector machine, random forest model, and generalized linear model, were utilized to further identify the optimal crosstalk genes between DN and NAFLD. An integrated network containing crosstalk genes, transcription factors, and associated pathways was developed.

**Results:**

Four gene expression datasets, including GSE66676 and GSE48452 for NAFLD and GSE30122 and GSE1009 for DN, were involved in this study. There were 80 common DEGs between the two diseases in total. The PPI network built with the 80 common genes included 77 nodes and 83 edges. Ten optimal crosstalk genes were selected by LASSO regression and Boruta algorithm, including *CD36, WIPI1, CBX7, FCN1, SLC35D2, CP, ZDHHC3, PTPN3, LPL*, and *SPP1*. Among these genes, LPL and SPP1 were the most significant according to NAFLD-transcription factor network. Five hundred twenty-nine nodes and 1,113 edges comprised the PPI network of activated pathway-gene. In addition, 14 common pathways of these two diseases were recognized using Gene Ontology (GO) analysis; among them, regulation of the lipid metabolic process is closely related to both two diseases.

**Conclusions:**

This study offers hints that NAFLD and DN have a common pathogenesis, and LPL and SPP1 are the most relevant crosstalk genes. Based on the common pathways and optimal crosstalk genes, our proposal carried out further research to disclose the etiology and pathology between the two diseases.

## Introduction

Diabetic nephropathy (DN) is a rigorous microvascular complication primarily associated with both type 1 and type 2 diabetes mellitus (T2DM) and has been the leading cause of end-stage renal disease (ESRD) worldwide (1–3). Both morbidity and mortality of DN have promptly increased around the world (1, 2, 4). Non-alcoholic fatty liver disease (NAFLD) has become pyramidally ordinary in parallel with the adding popularity of obesity and other components of the metabolic syndrome (5, 6). NAFLD is distinguished as the existence of fat storage ≥5% of liver weight with the absence of excessive alcohol consumption or secondary cause of liver diseases such as autoimmune hepatitis, hemochromatosis, and Wilson’s disease (3, 7–9). Being metabolic diseases, factors that contribute to NAFLD, such as diabetes, chronic inflammation, insulin resistance, and obesity, are also associated with the development of DN.

Several observational studies reported an impressive proportion that there were 70%–86% of patients with NAFLD also suffering from T2DM (6, 8, 10–12). Jia et al. (13) found that the cumulative incidence of DN in patients with NAFLD was much higher than those without it and that the liver fat content was positively correlated with increased occurrence of albuminuria and decreased glomerular filtration rate (GFR). Targher et al. (14) also found that the prevalence of diabetic retinopathy and chronic kidney disease (CKD) was significantly higher in patients with NAFLD. Previous epidemiological studies further suggested several contributors including metabolic syndrome, dysbiosis, unhealthy diets, platelet activation, and processes acting as the linking factors between NAFLD and CKD, which implied the potential correlations involved in the pathogenesis of liver and kidney disease (15). That NAFLD might be a risk factor for DN had been analyzed by some researchers (13). The relationship between NAFLD and DN seems rational and of clinical interest to some extent.

Based on the results of current observational studies, the potential contributions of genetic factors and protein–protein interactions (PPIs) on the correlation of NAFLD and DN should be further analyzed. In this study, bioinformatics analysis was used to disclose the crosstalk mechanisms between NAFLD and DN at the transcriptomic level. The mutual transcription characteristics would offer new insights into the common pathogenesis of NAFLD and DN. The purpose of this study is to recognize optimal crosstalk genes, participant pathways, and transcription factors (TFs). We hypothesize the existence of crosstalk genes between NAFLD and DN, then employed comprehensive bioinformatics and enrichment analyses to identify the common differentially expressed genes (DEGs) and the functional pathways of NAFLD and DN. At last, we identified 10 crosstalk genes, favoring the similarity between these two diseases.

## Materials and methods

### Study design and data collection

**Figure 1:**
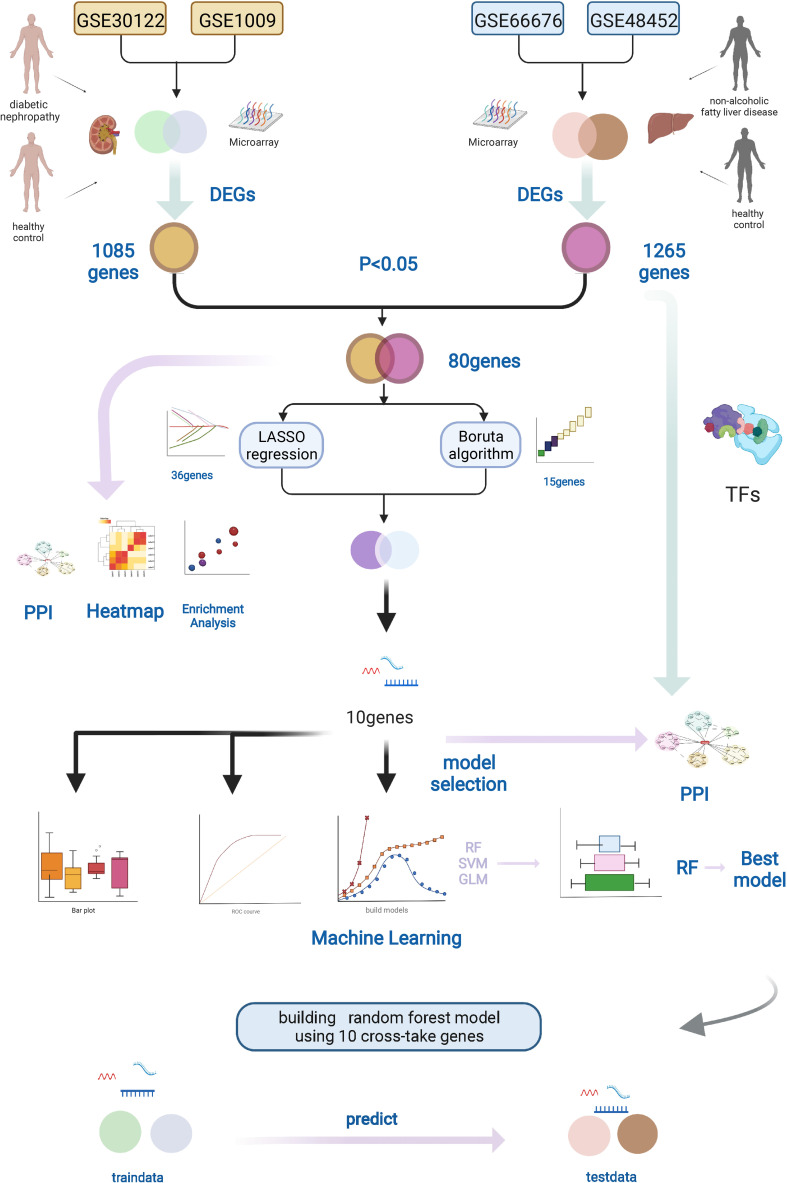
Workflow of this study. We downloaded the gene expression profiles of NAFLD and DN from the GEO database, including two NAFLD datasets (GSE66676 and GSE48452) and two DN datasets (GSE30122 and GSE1009). Datasets were merged, and DEGs were found by R software. The LASSO regression and Boruta algorithm were used to select the optional crosstalk genes. RF model was considered the best model to predict DN using the 10 crosstalk genes. In addition, protein–protein interaction (PPI) and functional enrichment analyses of the DEGs were performed. Graphic created with BioRender.com. NAFLD, nonalcoholic fatty liver disease; DN, diabetic nephropathy; GEO, Gene Expression Omnibus, DEGs, different expression genes.

We acquired microarray data from Gene Expression Omnibus (GEO) database (http://www.ncbi.nlm.nih.gov/geo/). After earnest review, four gene expression profiles (GSE66676 and GSE48452 were NAFLD datasets, and GSE30122 and GSE1009 were diabetic human kidney disease datasets, with no other complications) were selected. **Figure 1** shows the schematic of the research.

### Data procession and differentially expressed gene analysis

We combined two datasets for each disease to increase the sample size. R software (version 4.1.1; https://www.r-project.org/) and “BiocManager” packages were applied to analyze the data. The expression data from different datasets were normalized using the robust multi-array average and merged together, and the “sva” library was used for combating batch correction to remove batch effects. We then used the Linear Models for microarray data (“limma” package) to identify DEGs by comparing the expression values between NAFLD patients and control cases. Genes with *P* < 0.05 were considered DEGs. The same way was done in the diabetic kidney disease dataset. The “pheatmap” package was used to draw the heatmap of the DEGs in R software.

### Gene set enrichment analysis

In order to understand and interpret coordinate pathway-level changes in transcriptomics experiments represented under different conditions, the gene set enrichment analysis (GSEA) was used to determine whether there are statistically significant differences between the two groups as to a defined set of genes (16). R package “clusterProfiler” was used to perform GSEA. Gene sets “c7.all.v7.5.1.entrez” were downloaded from “Downloads (gsea-msigdb.org)“ website, and then R software was employed to retrieve systematic functional annotation information. *P* < 0.05 was the cutoff criterion. We examined the pathway-level changes for all DEGs in NAFLD and DN to find out whether there were reduplicative pathways.

### Immune infiltration analysis

The proportions of 22 kinds of immune cells, including naive B cells, memory B cells, plasma cells, CD8 T cells, naive CD4 T cells, CD4 resting memory T cells, activated memory CD4 T cells, helper follicular T cells, regulatory T cells (Tregs), delta gamma T cells, resting natural killer (NK) cells, activated natural killer (NK) cells, monocytes, macrophages M0, macrophages M1, macrophages M2, resting dendritic cells (DCs), active DCs, resting mast cells, activated mast cells, eosinophils, and neutrophils, were obtained, and CIBERSORT algorithm was utilized to analyze the gene expression data between NAFLD and DN.

We brought in all genes that were expressed in both NALFD and DN patients to explore the common ground based on 22 kinds of immune cells between the two diseases. The percentage of each kind of immune cell in the samples was calculated. Single-sample gene set enrichment analysis (ssGSEA) was employed to calculate the degree of penetration of 28 immune cell types on the grounds of the expression levels of genes in 28 published gene sets for immune cells (17).

### Identification of potential crosstalk genes and functional enrichment analysis

The potential crosstalk genes were identified as DN-related DEGs overlapped with the NAFLD-related ones. These crosstalk genes could have the potential ability of linking the pathogeneses of NAFLD and DN.

To further determine the biological features of potential crosstalk genes, Gene Ontology (GO) analysis was accomplished by “clusterProfiler“ of R Bioconductor packages. A classification method is offered by the “clusterProfiler“ packages to classify genes based on their projection at a specific level of the GO corpus and provide functions to calculate enrichment values for GO terms. The enriched function with *P* < 0.05 was considered a significant pathway. Based on this analysis method, we selected the top 20 GO biological processes. To identify the most significant clusters of the crosstalk genes, PPI network of crosstalk genes was constituted by STRING (STRING 11.5; Search Tool for the Retrieval of Interaction Gene; https://string-db.org/). Cytoscape (version 3.8.0) was used to visualize the PPI network.

### Identification of optimal diagnostic crosstalk genes

To better screen the risk crosstalk genes between NAFLD and DN, Boruta algorithm and least absolute shrinkage and selection operator (LASSO) regression were performed in R project. The LASSO regression was used to filter the best predictive features while fitting a generalized linear model (GLM) and avoiding overfitting. The Boruta employed a wrapper approach, built around a random forest (RF) classifier. After merging two NAFLD datasets, the expression values of potential crosstalk genes were extracted. The DEGs between NAFLD patients and healthy controls were reserved for feature selection, and the optimal crosstalk genes were initially recognized using the Boruta algorithm and LASSO regression. To narrow it down further, we combined the results of the two algorithms.

On the grounds of the optimal diagnostic crosstalk gene expression on the NAFLD merged dataset, we created the RF model, support vector machine (SVM) model, and GLM to pick out the best model. The response variable was the diagnosis of NAFLD or not, and the DEGs were used as explanatory variables. We then used the explain feature of “DALEX” package in R to find out which was the finest model among these three models aforementioned based on the plot of residual distribution.

### Development of the random forest model using optimal diagnostic crosstalk genes

After extracting the gene expression values of the filtered crosstalk genes that constitute the merged gene expression profile, the RF model with the gene expression value and sample type was built (NAFLD and healthy) to further confirm the diagnostic value of these crosstalk genes. The R package “randomForest” was applied to build the RF model. The “ComBat” method of “sva” packages in R project was performed to eliminate the batch effect. It is worth noting that the gene sample expression values were changed after a series of operations that are mentioned above, comparing previous gene expressions. Therefore, the primitive expression profile of the two datasets GSE66676 and GSE48452 was obtained. Afterward, the optimal crosstalk genes were confirmed by Boruta algorithm and LASSO regression, and then the expression values of the optimal crosstalk genes from the merged data were confirmed. We select the gene expression values of the filtered optimal genes to form the merged gene expression profile, and the RF models with the gene expression profile value and sample type were set up (NAFLD or healthy). The NAFLD merged data were input as training data, and the DN merged data were imported as testing data. The prediction effectiveness was determined by the accuracy rate of the test set.

### Transcription factor-adjusted and pathway analysis of the crosstalk genes

We downloaded TFs that regulate the target genes from TRRUST and ChEA3 databases, taking the intersection of TFs from these two databases. Based on the TF–target relationship, the NAFLD-related TF–target pairs were picked out, and the Cytoscape software was used to set up and visualize the TF–target gene interaction network.

In order to pick out activated pathways, the remarkably enriched pathways by the DEGs of NAFLD were screened. We selected the potential crosstalk pathways that may be the bridge of NAFLD and DN and obtained the genes functioning in each pathway. Finally, the Cytoscape software was used to construct the pathway–gene crosstalk network. For the purpose of confirming the functional TFs, which adjusted the crosstalk genes in the activated pathways, we picked out the crosstalk genes in the pathway–gene pairs and identified the NAFLD-related TFs and DN-related TFs. Moreover, 10 crosstalk genes were also included. Finally, the network of these four parts was created.

## Results

### Identification of differentially expressed genes and functional pathways by gene set enrichment analysis

A total of 215 study subjects were included in the current study. The mean age with standard deviation was 63.29 ± 14.61 years (DN patients) and 52.44 ± 12.90 years (healthy control) for the DN group and 45.92 ± 11.29 years (NAFLD patients) and 33.52 ± 8.82 years (healthy control) for the NAFLD group. The proportion of women was 66.5% and 78.9% for the DN and NAFLD groups, respectively. To identify DEGs between NAFLD and healthy controls, we recruited microarray expression profiles of GSE66676 and GSE48452 from the GEO database. After merging and normalizing the microarray data, 1,265 DEGs between NAFLD and healthy controls were selected by “limma” package (*P* < 0.05). Two DN datasets were also picked out from the GEO website, which was done in the same way. Finally, we got 1,265 DEGs in the NAFLD merged dataset and 1,085 DEGs in the DN merged dataset (heatmap shown in **Supplementary Figures S1A, B**). GSEA was implemented to reveal the functional similarity between the two diseases. All DEGs of each disease were contained in the GSEA using gene set “c7.all.v7.5.1.entrez.” As a result, three common pathways were identified in NAFLD and DN (**Supplementary Figures S2A, B**).

### Immune infiltration analysis

By employing the CIBERSORT algorithm, we investigated the similarity in immune infiltration between NAFLD patients and DN patients in 22 subpopulations of immune cells. The results acquired from NAFLD patients and DN patients were summarized by R software (**Supplementary Figure S3A**). The samples were screened according to *P* < 0.05, and the percentage of each kind of immune cell in the samples was calculated. As shown in **Supplementary Figure S3B**, there are no significant differences between NAFLD and DN tissue in most immune cells, such as macrophage M1, which were considered to be proinflammatory and promote inflammation (18). However, the DN tissue generally included a high ratio of naive CD4 T cells, delta gamma T cells, activated NK cells, and resting mast cells, while resting NK cells had the opposite trend of expression. In the ssGSEA (**Supplementary Figure S3C**), 17 immune cell subtypes, including activated B cell, NK T cell, immature B cell, effector memory CD8 T cell, and central memory CD4 T cell, demonstrated no significant expression differences between NAFLD and DN. However, Myeloid-derived suppressor cells (MDSC), memory B cells, regulatory T cells, T follicular helper cells, and Type 1 T helper cells showed higher expression in DN patients, while immature DCs were highly expressed in NAFLD patients. The consequences of the CIBERSORT algorithm and ssGSEA manifest that the two diseases are likely to have a similar immune infiltration environment, which laid the theoretical foundation to link them.

### Identifying crosstalk genes, Gene Ontology analysis, and construction of the protein–protein interaction network

**Figure 2:**
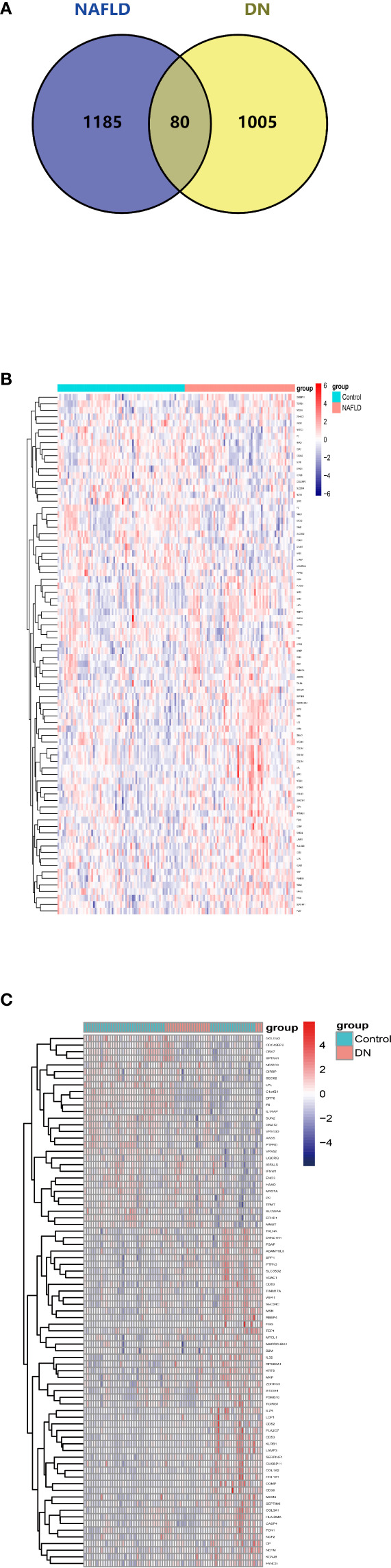
Venn diagram and expression level of common DEGs. **(A)** The intersection of DEGs in the NAFLD merged dataset and DN merged dataset from GEO contains 80 optimal crosstalk genes. The expression level of 80 common DEGs in the NAFLD merged dataset **(B)** and DN merged dataset **(C)**.

**Figure 3:**
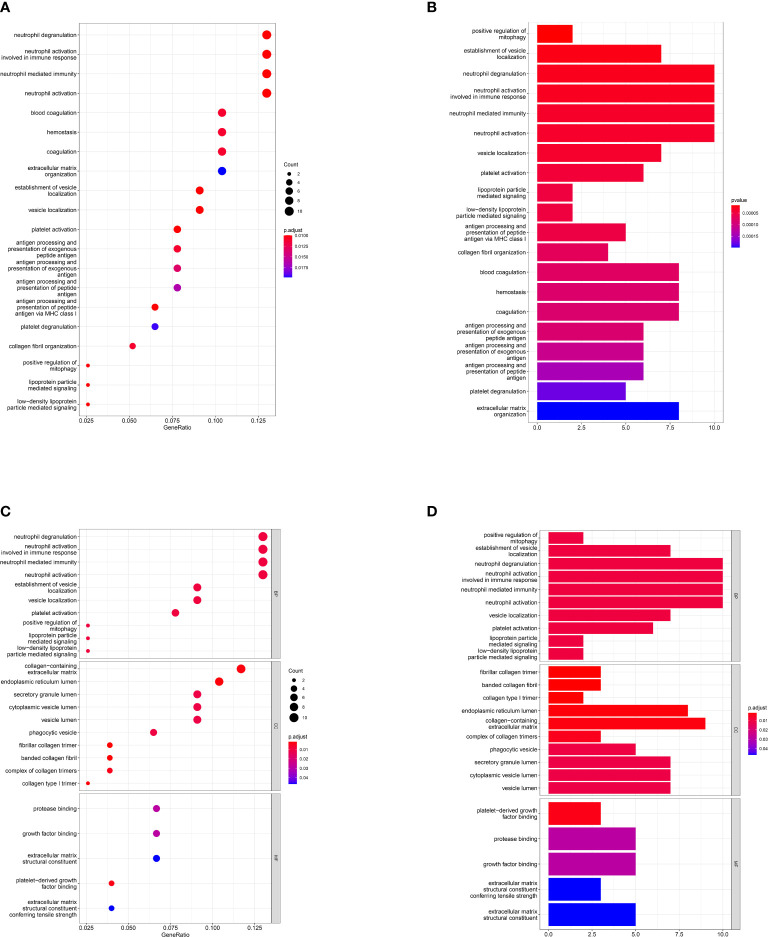
Gene Ontology pathway enrichment analysis. Result of GO pathway enrichment analysis of the 80 common DEGs, the dot plot **(A)** and bar plot **(B)**. Biological process (BP), cellular component (CC), and molecular function (MF) analysis results of 80 common DEGs, the dot plot **(C)** and bar plot **(D)**. X-axis represents the proportion of DEGs and Y-axis on behalf of the different categories. The size of the circle manifests the number of genes enriched in each category and different properties are denoted by the color of the circle.

After overlapping the DEGs of the two datasets, we finally got 80 crosstalk genes. The Venn diagram for the DEGs was given in **Figure 2A**. The heatmaps of common DEGs between NAFLD and DN were represented in **Figures 2B, C**. The GO analysis found that common DEGs were most intensively related to neutrophil-related pathways such as neutrophil degranulation, neutrophil activation involved in immune response, neutrophil-mediated immunity, and neutrophil activation. The detailed biological pathways in which DEGs were involved were shown in **Figures 3A–D**.

**Figure 4:**
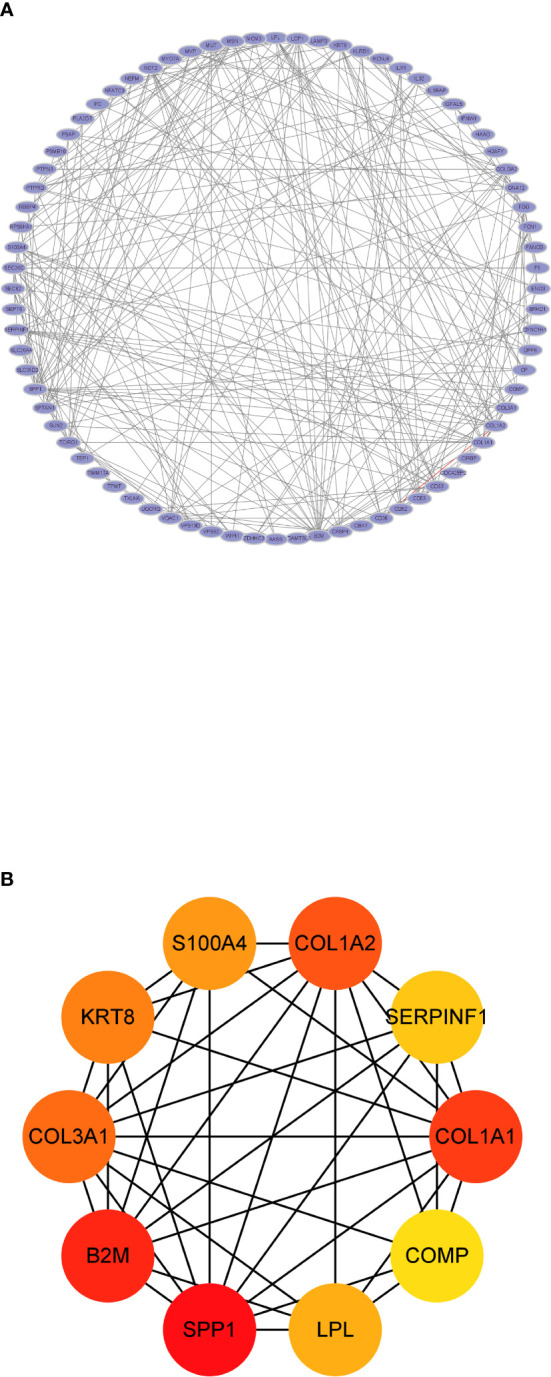
The protein–protein interaction analysis. **(A)** The PPI network analysis of the 80 common DEGs. **(B)** The hub genes identified by CytoHubba.

Seventy-seven nodes and 283 edges comprised the constructed PPI network of common DEGs (**Figure 4A**). The most significant module (score = 5.846) was recognized by MCODE, a plug-in of Cytoscape. CytoHubba was used to identify the hub genes (**Figure 4B**). SPP1 may be the key gene that contacts NAFLD and CKD, since it had the highest score in the biological network.

### Prediction of optimal crosstalk genes and building the machine learning model

**Figure 5:**
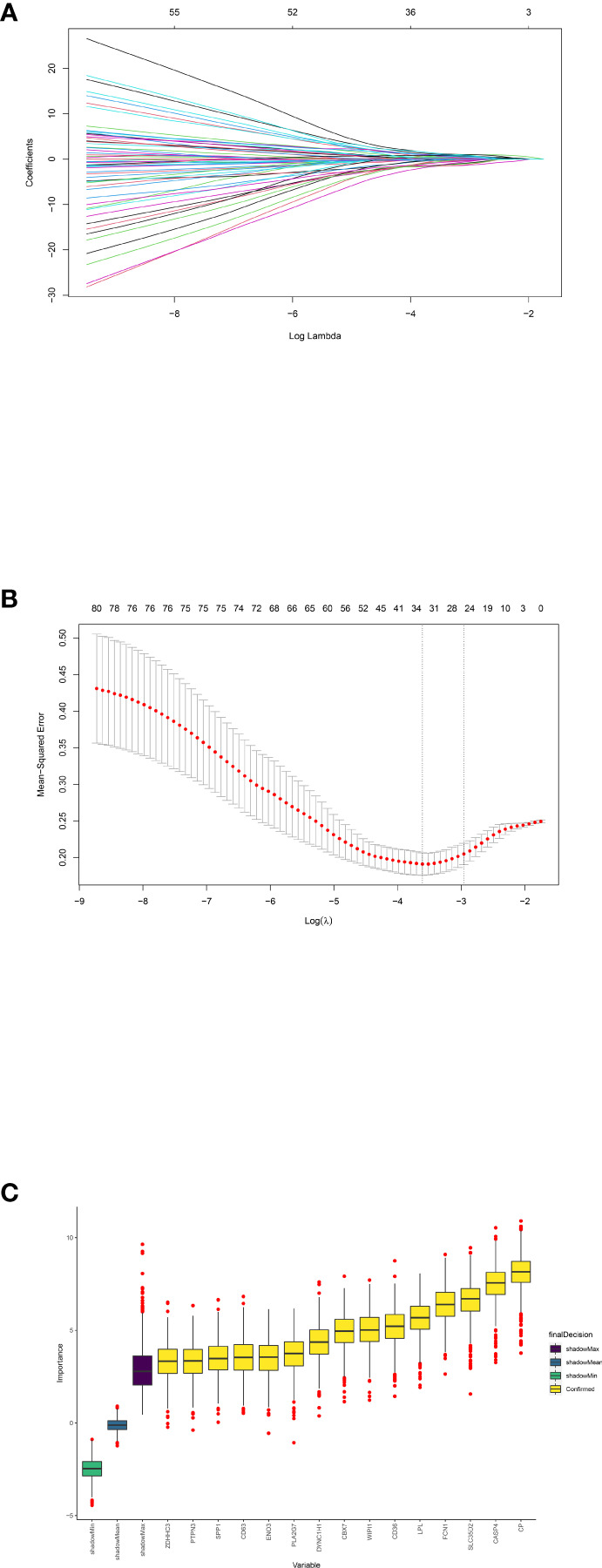
Feature selection. Crosstalk gene selection by LASSO regression **(A, B)** and Boruta algorithm **(C)**. **(A)** By using LASSO model to confirm the optimal genes, the partial likelihood deviance curve was plotted vs. log(lambda). Based on 1 SE of the minimum criteria (the 1-SE criteria) to draw dotted vertical lines. **(B)** Confirmed 34 genes with non-zero coefficients by optimal lambda. Panel **(A)** shows the coefficient profile plot produced against the log(lambda) sequence. **(C)** The 15 confirmed genes were indicated by the yellow box. X-axis represents selected genes. Y-axis represents the score of each gene.

We then extracted the expression data of the 80 genes from the NAFLD gene expression profile. Gene biomarkers were identified with the LASSO and Boruta algorithms. A total of 15 genes were finally selected (**Figures 5A–C**; **Supplementary Table S2**). Furthermore, the optimal crosstalk genes were identified by overlapping biomarkers derived from these two algorithms. We got 10 optimal crosstalk genes in the end.

**Figure 6:**
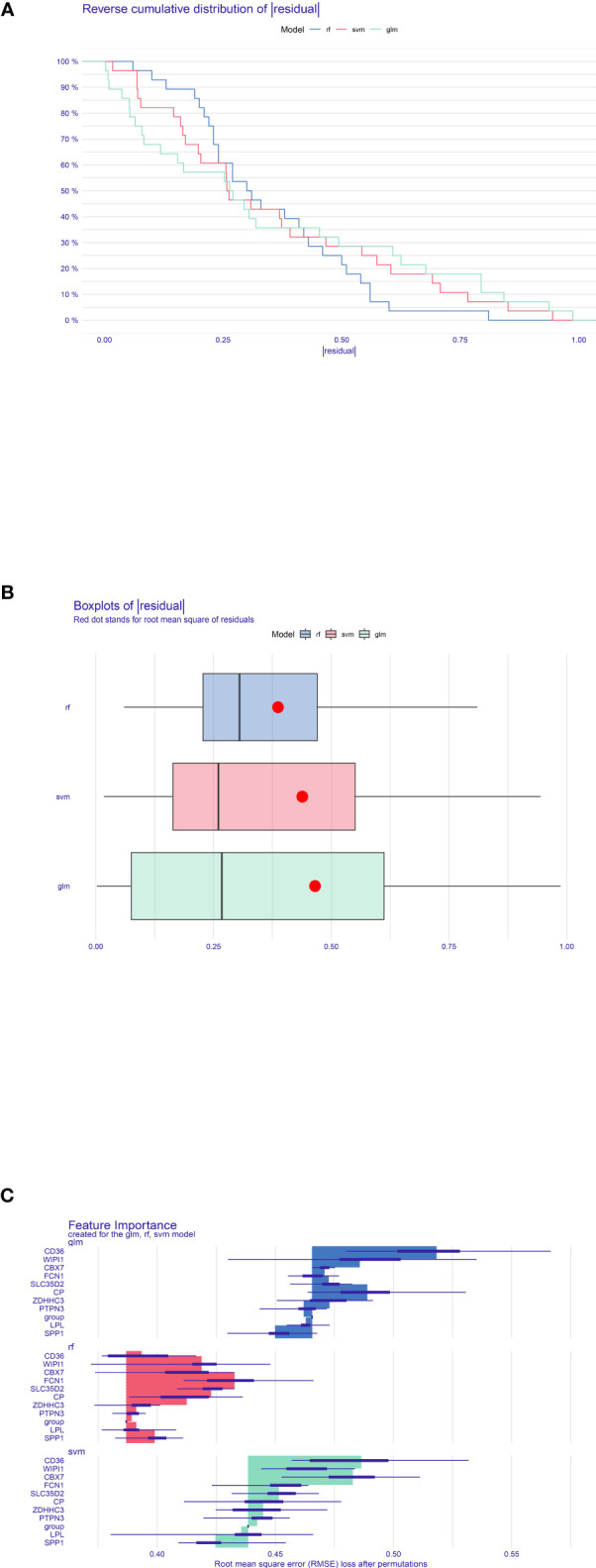
Construction and evaluation of RF model, SVM model, and GLM. **(A)** Accumulated residual distribution picture of the sample. **(B)** Boxplot of the residuals of the sample. The root mean square of the residuals was indicated by a red dot. **(C)** The significance of the variables in the three models.

To select and create the optimal prediction model, three models including SVM, RF, and GLM were created in light of the training NAFLD merged dataset. After that, the “DALEX” package’s explanatory feature in R was utilized to analyze the three aforementioned models. As shown in **Figures 6A–C**, which revealed the residual distribution, the RF model was confirmed as the best suitable model because it possesses the least sample residual. Ultimately, the expression of the 10 optimal crosstalk genes in NAFLD [*CD36, WIPI1, CBX7, FCN1, SLC35D2, CP, ZDHHC3, PTPN3*, lipoprotein lipase (LPL), and *SPP1*] was input to create the RF model. The gene expression profile of the 10 feature genes was also extracted from the DN merged dataset. Treating the NAFLD merged dataset as training data and the DN merged dataset as validation data, the predicted outcome of the RF model was shown in **Supplementary Table S1**. **Supplementary Figure S4** shows the importance of 10 genes in the RF model. The forecast performance of each gene in both NAFLD and DN was shown in **Supplementary Figure S5**. The area under the curve (AUC) values of LPL and SPP1 in DN were 86% and 80.1%, and the AUC values of LPL and SPP1 in NAFLD were 72.5% and 64.3%, respectively. **Supplementary Figure S6** showed the expression of the 10 genes in the two diseases.

### Transcription factor–gene regulation network

**Figure 7:**
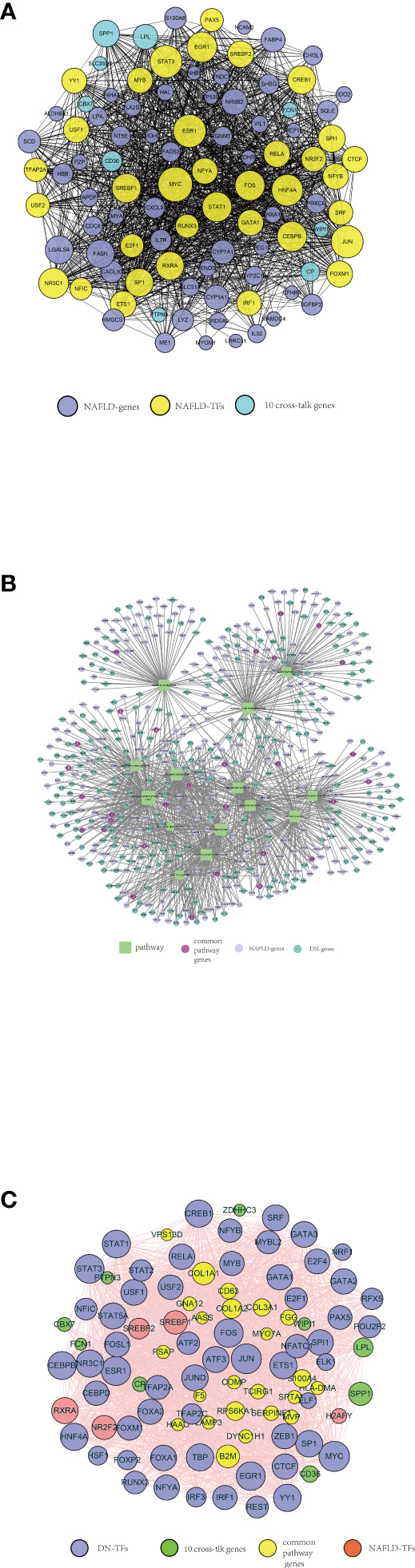
A series of protein–protein interaction (PPI) networks. **(A)** NAFLD-related TFs selected by TRRUST and ChEA3, indicated by yellow circles. The purple circles represent the top 50 significant differentially expressed genes in the NAFLD merged dataset. The optimal 10 crosstalk genes are indicated by the green circle. **(B)** Fourteen crucial pathways functioning in both NAFLD and DN, indicated by green squares. The purple-red circles represent the genes from each pathway both NAFLD-related and DN-related. The lilac circles represent the significant differentially expressed genes in the NAFLD merged dataset. The green circle represents the significant differentially expressed genes in the DN merged dataset. **(C)** DN-related TFs selected by TRRUST and ChEA3, indicated by purple circles. The green circles represent 10 optimal crosstalk genes. The genes from each pathway both NAFLD-related and DN-related were indicated by yellow circles. The pink circles represent NAFLD-related TFs.

We got a total of 35 mutual TFs, and the TF–target network was established as shown in **Figure 7A**. The optimal crosstalk genes with the highest degree were SPP1 and LPL and therefore potentially played a significant role in the TF–target network. **Supplementary Figure S7** showed the network between NAFLD TF–target pairs and DN–genes.

Ultimately, 14 crucial pathways, which may play a key role in the progress of NAFLD, were selected. In order to recognize the pathway crosstalk between NAFLD and DN, we established the pathway–gene crosstalk network. Five hundred twenty-nine nodes and 1,113 edges were included in the activated pathway–gene network (**Figure 7B**). To further explore the relationship between NAFLD and DN, the DN-related TF–target pairs and NAFLD-related TF–target pairs were extracted and the PPI was built. Meanwhile, the PPI between 10 crosstalk genes was obtained, then the activated TF–crosstalk gene network was established (**Figure 7C**). Consequently, we discovered that crosstalk genes were regulated by many TFs. The closeness of their relationship was indicated by the size of the circle. The highest degree among the 10 crosstalk genes remained to be SPP1 and LPL. DN-related TF–target pairs had a closer relationship with crosstalk genes.

## Discussion

The major outcome of this study was that bioinformatics analysis could expose crosstalk genes between NAFLD and DN. Accordingly, LPL and SPP1 were identified to be the most concerned genes; meanwhile, some participant pathways were identified. According to their expression values in each patient and forecast ability, the latent correlation of these genes was confirmed. The areas under the ROC curve of these two genes are higher than those of most genes (**Supplementary Figure S3**); furthermore, they have the highest degree in the TF–target network.

According to our results, the aberrant lipid and glucose metabolism plays an important role in the crosstalk between NAFLD and DN. The LPL gene, which belongs to the lipase gene family including hepatic lipase, endothelial lipase, and pancreatic lipase, could combine with lipoproteins and cell surface proteins concurrently, resulting in accumulation and uptake of lipoproteins (19, 20). In 2019, Teratani et al. (21) found that the expression of LPL was changed in hepatic stellate cells in NAFLD patients. Serum obesity-related factors, including interleukin-6, leptin, and free fatty acid (FA), could further affect its circulating level (21). NAFLD would even evolve to hepatocellular carcinoma (HCC) due to the aberrant activation of LPL, since it had great impact on HCC cell proliferation and lipid deposition (19).

On the other hand, LPL had also been proven to be associated with the development and progression of DN (20). A previous study has shown that DN rats have elevated levels of total cholesterol, triglycerides (TGs), and low-density lipoproteins (LDL), accompanied by significant changes in plasma LPL activity (22). When the activity of LPL is affected, it would consequently result in hypertriglyceridemia, which is a pivotal trait of nephrotic syndrome (23). Our previous studies also found that dyslipidemia was one of major risk factors for diabetic kidney disease (24, 25). In 2019, Al Shawaf et al. (23) found that the level of circulating ANGPTL4, an inhibitor of LPL, was significantly higher in DN patients compared with those in T2DM patients and healthy controls. Its expression was also positively correlated with serum creatinine and urinary albumin-to-creatinine ratio (23). These findings indicated that the suppressing efforts on LPL were increasing during the progression from DM to DN, which suggested an intervention target for the early prevention of the development of DN in DM patients.

Another crucial crosstalk gene we found was SPP1, which encodes osteopontin and is expressed in a variety of cells and tissues including endothelial cells, DCs, macrophages, and kidney (26, 27). Osteopontin is known as a regulator of hepatic stellate cell activation. Zhu et al. (28) found that the contribution of hepatocyte-derived osteopontin in NAFLD was capable of altering the liver microenvironment to potentiate fibrosis *via* a Notch-activated pathway. Notch-mediated osteopontin secretion in hepatocytes could directly activate hepatic stellate cells and cause excessive collagen deposition, despite hepatocellular injury. Furthermore, by performing chronic γ-secretase inhibitor treatment, liver Notch activity was decreased and hepatic stellate cell activation and liver fibrosis were reduced (28). In the progression of DN, Notch signaling pathway was activated following long-term exposure to hyperglycemia (29). The expression of constitutively active Notch intracellular domain in mature podocytes caused podocyte dedifferentiation, glomerulosclerosis, and apoptosis that substantially caused albuminuria and progressive renal failure (30, 31). By treating with γ-secretase inhibitors, the diabetes-induced glomerulosclerosis and podocyte injury could be prevented, which suggested that inhibiting the overactive Notch pathway in renal cells could be a potential plausible therapeutic approach (32).

Previous studies indicated that hyperglycemia would increase the expression of SPP1, which caused an elevated exposure of cells to proinflammatory cytokines and inflammation indicators including tumor necrosis factor α, transforming growth factor β, and interleukin-1 (33–36). In our current study, the immune filtration analysis also found that there were common expressions of macrophages and DCs between the NAFLD and DN groups, which suggested that inflammatory activations were involved in the crosstalk of the two diseases. It has been proven that in high-glucose conditions, the transcriptional activity of SPP1 was enhanced in proximal tubular epithelial cells (PTECs), which means that when T2DM occurred, the expression of SPP1 will increase and SPP1 promotes the occurrence and development of both diseases (37). Zhang et al. (38) also found that the SPP1 was negatively correlated with GFR in diabetic kidney disease patients. Some researchers believed that SPP1 could be the core target to treat diabetic kidney disease by using traditional Chinese medicine (39). SPP1 is also conjectured to function in the transformation of non-alcoholic steatohepatitis (NASH) to HCC like LPL (40).

In the enrichment analysis part, we found that 14 pathways were involved in the crosstalk between NAFLD and DN, one of which was “regulation of lipid metabolic process.” Several previous studies also demonstrated that the progression of DN was linked to serum lipid abnormalities and renal ectopic lipid accumulation (18, 41, 42). The proportion of kidney-absorbed LDL would be different when the activity of the LDL receptor changed; meanwhile, the expression of LDL receptor would be remarkably suppressed by cholesterol in podocytes (42). Lipid loading facilitates the phenotypic conversion of podocytes, which results in the disappearance of its epithelial features (43–45). Most DN patients performed albuminuria or macroproteinuria during the progression of disease. The albumin also acted as a vehicle for FAs in urine. Consequently, albuminuria may cause extensive accumulation of FAs and accelerate kidney injury in DN patients (46). By analyzing 34 DN patients and 12 healthy controls, Herman-Edelstein et al. (47) found a high degree of correlation between lipid metabolism and GFR. In the situation of continuing hyperglycemia in diabetic patients, TGs and FAs were accumulated (47). Ectopic lipid accumulation in non-adipose tissues, such as liver, kidney, heart, and pancreas, occurs because of raised serum TGs, FFAs, and modified cholesterol (41, 48–51), which appear to play a part in the pathogenesis of DN (52–54). This condition also seems to result in NAFLD. This suggests that diabetes also acts as a link between these two diseases. Consistent with this, obvious neutral lipid accumulation was found both in glomeruli and tubulointerstitium in diabetic kidneys (47). Two of the features of DN in electron microscope podocyte process effacement, interestingly, lipotoxicity and lipid cumulation, can lead to podocyte malfunction and apoptosis (55). Hence, it is reasonable for DN to see heavy lipid deposition (47).

A previous study found that the total counts of lipid droplets (LDs) decreased when kidney tissue was seriously fibrosed. This process was similar to the progression of NASH (56). Liver is the central organ of lipoprotein metabolism, since it takes part in the production of lipoprotein particles in all categories. It also plays a central role in the metabolism of TGs and cholesterol. Serum TGs and remnant cholesterol would be elevated when liver function is impaired. Then, it comes with altered glucose metabolism and insulin resistance, which are believed to be hallmarks of NAFLD (57). NAFLD occurs at the time of excessive intake of FAs and TGs from the circulation. Unbalanced lipid metabolism is also related to NAFLD advancement from steatosis to NASH; moreover, alterations in liver and serum lipidomic signatures are excellent indicators of NAFLD’s development and progression (58).

Both NAFLD and DN were considered to be affected by chronic inflammation progression, especially in individuals with abnormal serum glucose and lipid concentration (7, 12, 59). In our current study, we also found that there were aberrant proportions and expression levels of immune indicators in both diseases. There were a total of 7 and 17 kinds of immune cells performing higher proportion and expression levels, respectively. Most of them showed no significant differences between NAFLD and DN groups, which indicated the mutual mechanism in the two diseases. Enrichment analysis further demonstrated that several common DEGs were enriched in immune-related functions, including neutrophil activation, neutrophil-mediated immunity, and positive regulation of mitophagy.

Liver-mediated lipid changes are associated with the severity of proteinuria (60). Similarly, the diagnosis of DN refers to the appearance of specific pathologic structural alongside functional changes in the kidney of patients with DM, one of which is proteinuria (61). Renal injury is generally non-reversible on the condition that albuminuria persistently occurs (13). Various mechanisms, such as poor plasma glucose control, activation of sympathetic nervous system, and insulin resistance both in liver and kidney contribute coordinately to the advancement of kidney diseases (62, 63). Metabolic syndrome is a significant contributor to the evolution of chronic kidney dysfunction (64, 65). It has already been confirmed that lipid accumulation is strongly associated with inflammatory stress in the kidney. Through perturbing the LDL receptor pathway and induced phenotypic change and dysfunction in podocytes, inflammation induces lipid accumulation (42). Obesity is closely associated with these two diseases, which have been considered as a risk factor for both NAFLD and DN (9, 66, 67). Obesity, T2DM, and NAFLD can not only facilitate systemic insulin resistance but also boost the accumulation of hepatic fat and impairment of glucose metabolism (3, 66); in the meantime, insulin resistance can stimulate hepatic macrophages. T2DM often causes chronic hyperinsulinemia, which plays a vital role in liver metabolism abnormalities (41). The possibility that kidney dysfunction occurs was remarkably higher in patients with NAFLD based on two cross-sectional studies (14, 68). There is an assumption that along with the advancement of NAFLD into NASH, diabetic kidney diseases would occur (69). With the progression of NASH, metabolic disorders, for example, dyslipidemia, insulin resistance, and glucose intolerance, would collectively advance the renin-angiotensin (RAS) system system and influence nitric oxide formation (70–72), which can facilitate the progression of DN. Simultaneously, a mechanism in NASH adjusted by liver-derived inflammatory mediators and oxidative stress, which boosts the free proinflammatory, procoagulant, pro-oxidant, and profibrogenic factors from the liver, participates in the development of DN (69, 73–75). Renal hemodynamics may be influenced because of activation of the sympathetic nervous system, hence conducing to the onset or deterioration of kidney diseases (3, 63). Therefore, based on these common risk factors, it is reasonable to conjecture the interlink between NAFLD and DN, and early detection and treatment of NAFLD may be of clinical significance for the intervention of DN.

Altogether, this study identified two major crosstalk genes and relevant shared pathways, which emphasize the comparability and underlying relationship between NAFLD and DN. It is reasonable to consider that NAFLD patients are vulnerable to get and develop kidney dysfunction caused by T2DM and insulin insistence. Despite the credibility of such a probability interlink, more research needs to be done to reveal the potential mechanism of these two diseases. Nevertheless, there exist several limitations in our study. Firstly, data on survival times and outcomes are lacking. Therefore, the effect of these crosstalk genes on survival could not be tested. Secondly, the integration of different gene expression datasets might be biased because of discrepancies in the experimental setting for each dataset. Ideally, unnecessary bias should be avoided by making sure all gene expression data have the same experimental settings. In addition, it was a retrospective study. For the purpose of averting analysis bias associated with retrospective studies, a prospective study is recommended to be conducted. Lastly, the current study is entirely ground on computer analyses, thus the validation analysis based on wet-lab will be encouraged to confirm these crosstalk genes we found.

## Conclusion

To the best of our knowledge, this is the first study of crosstalk mechanisms between NAFLD and DN using bioinformatics analysis, identifying common immune and TF-related mechanisms. LPL and SPP1 are the most relevant crosstalk genes in our study, which suggest that NAFLD and DN may have a common pathogenesis.

## Data availability statement

The original contributions presented in the study are included in the article/**Supplementary Material**. Further inquiries can be directed to the corresponding authors.

## Author contributions

QY, ZZ, JYD, and DL designed the study, collected the data, analyzed the data, and wrote the manuscript. ZL, JCD, SP, and JL reviewed and revised the manuscript. All authors contributed to the article and approved the submitted version.

## Funding

This work was supported by the Young Scientists Fund of the National Natural Science Foundation of China (Grant No. 82103916), General Program of the National Science Foundation of China General Project (No. 81970633).

## Conflict of interest

The authors declare that the research was conducted in the absence of any commercial or financial relationships that could be construed as a potential conflict of interest.

## Publisher’s note

All claims expressed in this article are solely those of the authors and do not necessarily represent those of their affiliated organizations, or those of the publisher, the editors and the reviewers. Any product that may be evaluated in this article, or claim that may be made by its manufacturer, is not guaranteed or endorsed by the publisher.

## Supplementary material

Supplementary Figure 1 The functional similarity between NAFLD and DN. **(A)** Comparison of functional enrichment of DN and healthy controls. **(B)** Comparison of functional enrichment of NAFLD and healthy controls.

Supplementary Figure 2 The expression level of all DEGs The expression level of all DEGs in NAFLD merged dataset **(A)** and DN merged dataset **(B)**.

Supplementary Figure 3 Immune characteristics of NAFLD and DN Comparison of immune characteristics between NAFLD and DN. **(A)** An intuitive picture of the percentage of the 22 immune cells in NAFLD patients and DN patients. **(B)** The proportion of 22 immune cells in two diseases. **(C)** The expression of 28 immune cells in two diseases.

Supplementary Figure 4 Rank the importance of the RF model.

Supplementary Figure 5 The predictive efficiency of the 10 crosstalk genes in NAFLD **(A)** and DN **(B)**.

Supplementary Figure 6 The expression of the 10 crosstalk genes in NAFLD **(A)** and DN **(B)**.

Supplementary Figure 7 The protein-protein interaction network of the NAFLD related TFs and the top 180 significant differentially expressed genes in DN merged dataset.

## References

[1] Kato M Natarajan R . Diabetic nephropathy–emerging epigenetic mechanisms. Nat Rev Nephrol (2014) 10(9):517–30. doi: 10.1038/nrneph.2014.116 PMC6089507 25003613

[2] Xiong Y Zhou L . The signaling of cellular senescence in diabetic nephropathy. Oxid Med Cell Longev (2019) 2019:7495629. doi: 10.1155/2019/7495629 31687085 PMC6794967

[3] Wang T-Y Wang R-F Bu Z-Y Targher G Byrne CD Sun D-Q . Association of metabolic dysfunction-associated fatty liver disease with kidney disease. Nat Rev Nephrol (2022) 18(4):259–68. doi: 10.1038/s41581-021-00519-y 35013596

[4] Bell S Fletcher EH Brady I Looker HC Levin D Joss N . End-stage renal disease and survival in people with diabetes: a national database linkage study. QJM (2015) 108(2):127–34. doi: 10.1093/qjmed/hcu170 PMC4309927 25140030

[5] Neuschwander-Tetri BA . Non-alcoholic fatty liver disease. BMC Med (2017) 15(1):45. doi: 10.1186/s12916-017-0806-8 28241825 PMC5330146

[6] Arslan MS Turhan S Dincer I Mizrak D Corapcioglu D Idilman R . A potential link between endothelial function, cardiovascular risk, and metabolic syndrome in patients with non-alcoholic fatty liver disease. Diabetol Metab Syndr (2014) 14(6):109. doi: 10.1186/1758-5996-6-109 PMC4424578 25960770

[7] Wijarnpreecha K Thongprayoon C Boonpheng B Panjawatanan P Sharma K Ungprasert P . Nonalcoholic fatty liver disease and albuminuria: a systematic review and meta-analysis. Eur J Gastroenterol Hepatol (2018) 30(9):986–94. doi: 10.1097/MEG.0000000000001169 29787418

[8] Heidari Z Gharebaghi A . Prevalence of non alcoholic fatty liver disease and its association with diabetic nephropathy in patients with type 2 diabetes mellitus. J Clin Diagn Res (2017) 11(5):OC04–OC7. doi: 10.7860/JCDR/2017/25931.9823 PMC5483726 28658824

[9] Musso G Gambino R Cassader M Pagano G . Meta-analysis: natural history of non-alcoholic fatty liver disease (NAFLD) and diagnostic accuracy of non-invasive tests for liver disease severity. Ann Med (2011) 43(8):617–49. doi: 10.3109/07853890.2010.518623 21039302

[10] Targher G Chonchol M Bertolini L Rodella S Zenari L Lippi G . Increased risk of CKD among type 2 diabetics with nonalcoholic fatty liver disease. J Am Soc Nephrol (2008) 19(8):1564–70. doi: 10.1681/ASN.2007101155 PMC2488256 18385424

[11] Tolman KG Fonseca V Dalpiaz A Tan MH . Spectrum of liver disease in type 2 diabetes and management of patients with diabetes and liver disease. Diabetes Care (2007) 30(3):734–43. doi: 10.2337/dc06-1539 17327353

[12] Targher G Tessari R Bertolini L . Prevalence of Nonalcoholic fatty liver disease and its association wuth cardiovascular disease among type 2 diabetic patients. Diabetes Care (2007) 30: 734–43. doi: 10.2337/dc06-porting 17277038

[13] Jia G Di F Wang Q Shao J Gao L Wang L . Non-alcoholic fatty liver disease is a risk factor for the development of diabetic nephropathy in patients with type 2 diabetes mellitus. PloS One (2015) 10(11):e0142808. doi: 10.1371/journal.pone.0142808 26566287 PMC4643958

[14] Targher G Bertolini L Rodella S Zoppini G Lippi G Day C . Non-alcoholic fatty liver disease is independently associated with an increased prevalence of chronic kidney disease and proliferative/laser-treated retinopathy in type 2 diabetic patients. Diabetologia (2008) 51(3):444–50. doi: 10.1007/s00125-007-0897-4 18058083

[15] Byrne CD Targher G . NAFLD as a driver of chronic kidney disease. J Hepatol (2020) 72(4):785–801. doi: 10.1016/j.jhep.2020.01.013 32059982

[16] Zeng M Liu J Yang W Zhang S Liu F Dong Z . Multiple-microarray analysis for identification of hub genes involved in tubulointerstial injury in diabetic nephropathy. J Cell Physiol (2019). doi: 10.1002/jcp.28313 30761531

[17] Yan G An Y Xu B Wang N Sun X Sun M . Potential impact of ALKBH5 and YTHDF1 on tumor immunity in colon adenocarcinoma. Front Oncol (2021) 11:670490. doi: 10.3389/fonc.2021.670490 34079761 PMC8165310

[18] Yuan F Zhang Q Dong H Xiang X Zhang W Zhang Y . Effects of des-acyl ghrelin on insulin sensitivity and macrophage polarization in adipose tissue. J Transl Int Med (2021) 9(2):84–97. doi: 10.2478/jtim-2021-0025 34497748 PMC8386331

[19] Wu Z Ma H Wang L Song X Zhang J Liu W . Tumor suppressor ZHX2 inhibits NAFLD-HCC progression *via* blocking LPL-mediated lipid uptake. Cell Death Differ (2020) 27(5):1693–708. doi: 10.1038/s41418-019-0453-z PMC7206072 31740790

[20] Mead JR Irvine SA Ramji DP . Lipoprotein lipase: structure, function, regulation, and role in disease. J Mol Med (Berl) (2002) 80(12):753–69. doi: 10.1007/s00109-002-0384-9 12483461

[21] Teratani T Tomita K Furuhashi H Sugihara N Higashiyama M Nishikawa M . Lipoprotein lipase up-regulation in hepatic stellate cells exacerbates liver fibrosis in nonalcoholic steatohepatitis in mice. Hepatol Commun (2019) 3(8):1098–112. doi: 10.1002/hep4.1383 PMC6671781 31388630

[22] Kandasamy N Ashokkumar N . Renoprotective effect of myricetin restrains dyslipidemia and renal mesangial cell proliferation by the suppression of sterol regulatory element binding proteins in an experimental model of diabetic nephropathy. Eur J Pharmacol (2014) 743:53–62. doi: 10.1016/j.ejphar.2014.09.014 25240712

[23] Al Shawaf E Abu-Farha M Devarajan S Alsairafi Z Al-Khairi I Cherian P . ANGPTL4: A predictive marker for diabetic nephropathy. J Diabetes Res (2019) 2019:4943191. doi: 10.1155/2019/4943191 31772941 PMC6854918

[24] Duan JY Duan GC Wang CJ Liu DW Qiao YJ Pan SK . Prevalence and risk factors of chronic kidney disease and diabetic kidney disease in a central Chinese urban population: a cross-sectional survey. BMC Nephrol (2020) 21(1):115. doi: 10.1186/s12882-020-01761-5 32245423 PMC7118942

[25] Duan J Wang C Liu D Qiao Y Pan S Jiang D . Prevalence and risk factors of chronic kidney disease and diabetic kidney disease in Chinese rural residents: a cross-sectional survey. Sci Rep (2019) 9(1):10408. doi: 10.1038/s41598-019-46857-7 31320683 PMC6639314

[26] Ashizawa N Graf K Do YS Nunohiro T Giachelli CM Meehan WP . Osteopontin is produced by rat cardiac fibroblasts and mediates a II -induced DNA synthesis and collagen gel contraction. J Clin Invest (1996) 98(10):2218–27. doi: 10.1172/JCI119031 PMC507670 8941637

[27] Murry CE Giachelli CM Schwartz SM Vrackot R . Macrophages express osteopontin during repair of myocardial necrosis. Am J Pathol (1994) 145(6):1450–62. PMC1887495 7992848

[28] Zhu C Kim K Wang X Bartolome A Salomao M Dongiovanni P . Hepatocyte notch activation induces liver fibrosis in nonalcoholic steatohepatitis. Sci Transl Med (2018) 10(468):eaat0344. doi: 10.1126/scitranslmed.aat0344 30463916 PMC6822168

[29] Lin CL Wang FS Hsu YC Chen CN Tseng MJ Saleem MA . Modulation of notch-1 signaling alleviates vascular endothelial growth factor-mediated diabetic nephropathy. Diabetes (2010) 59(8):1915–25. doi: 10.2337/db09-0663 PMC2911050 20522599

[30] Niranjan T Bielesz B Gruenwald A Ponda MP Kopp JB Thomas DB . The notch pathway in podocytes plays a role in the development of glomerular disease. Nat Med (2008) 14(3):290–8. doi: 10.1038/nm1731 18311147

[31] Waters AM Wu MYJ Onay T Scutaru J Liu J Lobe CG . Ectopic notch activation in developing podocytes causes glomerulosclerosis. J Am Soc Nephrol (2008) 19(6):1139–57. doi: 10.1681/ASN.2007050596 PMC2396929 18337488

[32] Bonegio R Susztak K . Notch signaling in diabetic nephropathy. Exp Cell Res (2012) 318(9):986–92. doi: 10.1016/j.yexcr.2012.02.036 PMC3677813 22414874

[33] Guo H Cai CQ Schroeder RA Kuo PC . Osteopontin is a negative feedback regulator of nitric oxide synthesis in murine macrophages. J Immunol (2001) 166(2):1079–86. doi: 10.4049/jimmunol.166.2.1079 11145688

[34] Ricardo SD Franzoni DF Roesener CD Crisman JM Diamond JR . Angiotensinogen and AT1 antisense inhibition of osteopontin translation in rat proximal tubular cells. Am J Physiol Renal Physiol (2000) 278(5):F708–16. doi: 10.1152/ajprenal.2000.278.5.F708 10807582

[35] Hullinger TG Pan Q Viswanathan HL Somerman MJ . TGFbeta and BMP-2 activation of the OPN promoter: roles of smad- and hox-binding elements. Exp Cell Res (2001) 262(1):69–74. doi: 10.1006/excr.2000.5074 11120606

[36] Sodhi CP Phadke SA Batlle D Sahai A . Hypoxia and high glucose cause exaggerated mesangial cell growth and collagen synthesis: role of osteopontin. Am J Physiol Renal Physiol (2001) 280(4):F667–74. doi: 10.1152/ajprenal.2001.280.4.F667 11249858

[37] Shirakawa K Sano M . Sodium-glucose Co-transporter 2 inhibitors correct metabolic maladaptation of proximal tubular epithelial cells in high-glucose conditions. Int J Mol Sci (2020) 21(20). doi: 10.3390/ijms21207676 PMC7589591 33081406

[38] Zhang Y Li W Zhou Y . Identification of hub genes in diabetic kidney disease *via* multiple-microarray analysis. Ann Transl Med (2020) 8(16):997. doi: 10.21037/atm-20-5171 32953797 PMC7475500

[39] Xing L Xing W Guo H . [Exploring the therapeutic mechanism of longqi fang for diabetic kidney disease based on network pharmacology and verification in rats]. Nan Fang Yi Ke Da Xue Xue Bao (2022) 42(2):171–80. doi: 10.12122/j.issn.1673-4254.2022.02.02 PMC8983373 35365440

[40] Wang Z Zhao Z Xia Y Cai Z Wang C Shen Y . Potential biomarkers in the fibrosis progression of nonalcoholic steatohepatitis (NASH). J Endocrinol Invest (2022) 45(7):1379–92. doi: 10.1007/s40618-022-01773-y 35226336

[41] Opazo-Rios L Mas S Marin-Royo G Mezzano S Gomez-Guerrero C Moreno JA . Lipotoxicity and diabetic nephropathy: Novel mechanistic insights and therapeutic opportunities. Int J Mol Sci (2020) 21(7). doi: 10.3390/ijms21072632 PMC7177360 32290082

[42] Zhang Y Ma KL Liu J Wu Y Hu ZB Liu L . Inflammatory stress exacerbates lipid accumulation and podocyte injuries in diabetic nephropathy. Acta Diabetol (2015) 52(6):1045–56. doi: 10.1007/s00592-015-0753-9 25896009

[43] Reidy K Susztak K . Epithelial-mesenchymal transition and podocyte loss in diabetic kidney disease. Am J Kidney Dis (2009) 54(4):590–3. doi: 10.1053/j.ajkd.2009.07.003 PMC2761212 19781451

[44] Li Y Kang YS Dai C Kiss LP Wen X Liu Y . Epithelial-to-mesenchymal transition is a potential pathway leading to podocyte dysfunction and proteinuria. Am J Pathol (2008) 172(2):299–308. doi: 10.2353/ajpath.2008.070057 18202193 PMC2312375

[45] Yamaguchi Y Iwano M Suzuki D Nakatani K Kimura K Harada K . Epithelial-mesenchymal transition as a potential explanation for podocyte depletion in diabetic nephropathy. Am J Kidney Dis (2009) 54(4):653–64. doi: 10.1053/j.ajkd.2009.05.009 19615802

[46] Weinberg JM . Lipotoxicity. Kidney Int (2006) 70(9):1560–6. doi: 10.1038/sj.ki.5001834 16955100

[47] Herman-Edelstein M Scherzer P Tobar A Levi M Gafter U . Altered renal lipid metabolism and renal lipid accumulation in human diabetic nephropathy. J Lipid Res (2014) 55(3):561–72. doi: 10.1194/jlr.P040501 PMC3934740 24371263

[48] Sharma S Adrogue JV Golfman L Uray I Lemm J Youker K . Intramyocardial lipid accumulation in the failing human heart resembles the lipotoxic rat heart. FASEB J (2004) 18(14):1692–700. doi: 10.1096/fj.04-2263com 15522914

[49] Schulze PC . Myocardial lipid accumulation and lipotoxicity in heart failure. J Lipid Res (2009) 50(11):2137–8. doi: 10.1194/jlr.R001115 PMC2759818 19687505

[50] Marfella R Di Filippo C Portoghese M Barbieri M Ferraraccio F Siniscalchi M . Myocardial lipid accumulation in patients with pressure-overloaded heart and metabolic syndrome. J Lipid Res (2009) 50(11):2314–23. doi: 10.1194/jlr.P900032-JLR200 PMC2759838 19470430

[51] Belopolsky Y Khan MQ Sonnenberg A Davidson DJ Fimmel CJ . Ketogenic, hypocaloric diet improves nonalcoholic steatohepatitis. J Transl Int Med (2020) 8(1):26–31. doi: 10.2478/jtim-2020-0005 32435609 PMC7227162

[52] Proctor G Jiang T Iwahashi M Wang Z Li J Levi M . Regulation of renal fatty acid and cholesterol metabolism, inflammation, and fibrosis in akita and OVE26 mice with type 1 diabetes. Diabetes (2006) 55(9):2502–9. doi: 10.2337/db05-0603 16936198

[53] Kim HJ Moradi H Yuan J Norris K Vaziri ND . Renal mass reduction results in accumulation of lipids and dysregulation of lipid regulatory proteins in the remnant kidney. Am J Physiol Renal Physiol (2009) 296(6):F1297–306. doi: 10.1152/ajprenal.90761.2008 PMC2692452 19357177

[54] Bobulescu IA . Renal lipid metabolism and lipotoxicity. Curr Opin Nephrol Hypertens (2010) 19(4):393–402. doi: 10.1097/MNH.0b013e32833aa4ac 20489613 PMC3080272

[55] Nosadini R Tonolo G . Role of oxidized low density lipoproteins and free fatty acids in the pathogenesis of glomerulopathy and tubulointerstitial lesions in type 2 diabetes. Nutr Metab Cardiovasc Dis (2011) 21(2):79–85. doi: 10.1016/j.numecd.2010.10.002 21186102

[56] Nagaya T Tanaka N Suzuki T Sano K Horiuchi A Komatsu M . Down-regulation of SREBP-1c is associated with the development of burned-out NASH. J Hepatol (2010) 53(4):724–31. doi: 10.1016/j.jhep.2010.04.033 20655124

[57] Deprince A Haas JT Staels B . Dysregulated lipid metabolism links NAFLD to cardiovascular disease. Mol Metab (2020) 42:101092. doi: 10.1016/j.molmet.2020.101092 33010471 PMC7600388

[58] Mato JM Alonso C Noureddin M Lu SC . Biomarkers and subtypes of deranged lipid metabolism in non-alcoholic fatty liver disease. World J Gastroenterol (2019) 25(24):3009–20. doi: 10.3748/wjg.v25.i24.3009 PMC6603806 31293337

[59] Lv WS Sun RX Gao YY Wen JP Pan RF Li L . Nonalcoholic fatty liver disease and microvascular complications in type 2 diabetes. World J Gastroenterol (2013) 19(20):3134–42. doi: 10.3748/wjg.v19.i20.3134 PMC3662955 23716995

[60] Vaziri ND . Disorders of lipid metabolism in nephrotic syndrome: mechanisms and consequences. Kidney Int (2016) 90(1):41–52. doi: 10.1016/j.kint.2016.02.026 27165836 PMC5812444

[61] Umanath K Lewis JB . Update on diabetic nephropathy: Core curriculum 2018. Am J Kidney Dis (2018) 71(6):884–95. doi: 10.1053/j.ajkd.2017.10.026 29398179

[62] Targher G Byrne CD . Non-alcoholic fatty liver disease: an emerging driving force in chronic kidney disease. Nat Rev Nephrol (2017) 13(5):297–310. doi: 10.1038/nrneph.2017.16 28218263

[63] Spoto B Pisano A Zoccali C . Insulin resistance in chronic kidney disease: a systematic review. Am J Physiol Renal Physiol (2016) 311(6):F1087–F108. doi: 10.1152/ajprenal.00340.2016 27707707

[64] Chen J Muntner P Hamm LL Fonseca V Batuman V Whelton PK . Insulin resistance and risk of chronic kidney disease in nondiabetic US adults. J Am Soc Nephrol (2003) 14(2):469–77. doi: 10.1097/01.ASN.0000046029.53933.09 12538749

[65] Chen J . The metabolic syndrome and chronic kidney disease in U.S. adults. Ann Intern Med (2004) 140(3):167–74. doi: 10.7326/0003-4819-140-3-200402030-00007 14757614

[66] Mantovani A Petracca G Beatrice G Tilg H Byrne CD Targher G . Non-alcoholic fatty liver disease and risk of incident diabetes mellitus: an updated meta-analysis of 501 022 adult individuals. Gut (2021) 70(5):962–9. doi: 10.1136/gutjnl-2020-322572 32938692

[67] Wan H Wang Y Xiang Q Fang S Chen Y Chen C . Associations between abdominal obesity indices and diabetic complications: Chinese visceral adiposity index and neck circumference. Cardiovasc Diabetol (2020) 19. doi: 10.1186/s12933-020-01095-4 PMC7395356 32736628

[68] Targher G Bertolini L Chonchol M Rodella S Zoppini G Lippi G . Non-alcoholic fatty liver disease is independently associated with an increased prevalence of chronic kidney disease and retinopathy in type 1 diabetic patients. Diabetologia (2010) 53(7):1341–8. doi: 10.1007/s00125-010-1720-1 20369224

[69] Saito H Tanabe H Kudo A Machii N Higa M Yamaguchi S . High FIB4 index is an independent risk factor of diabetic kidney disease in type 2 diabetes. Sci Rep (2021) 11(1):11753. doi: 10.1038/s41598-021-88285-6 34083571 PMC8175689

[70] Cohen DE Fisher EA . Lipoprotein metabolism, dyslipidemia, and nonalcoholic fatty liver disease. Semin Liver Dis (2013) 33(4):380–8. doi: 10.1055/s-0033-1358519 PMC3988578 24222095

[71] Goh GB Pagadala MR Dasarathy J Unalp-Arida A Sargent R Hawkins C . Renin-angiotensin system and fibrosis in non-alcoholic fatty liver disease. Liver Int (2015) 35(3):979–85. doi: 10.1111/liv.12611 24905085

[72] Targher G . Risk of cardiovascular disease in patients with nonalcoholic fatty liver disease. Massachusetts Med Soc (2010) 363(14):1341–50. doi: 10.1056/NEJMra0912063 20879883

[73] Anders HJ Huber TB Isermann B Schiffer M . CKD in diabetes: diabetic kidney disease versus nondiabetic kidney disease. Nat Rev Nephrol (2018) 14(6):361–77. doi: 10.1038/s41581-018-0001-y 29654297

[74] Musso G Cassader M Cohney S De Michieli F Pinach S Saba F . Fatty liver and chronic kidney disease: Novel mechanistic insights and therapeutic opportunities. Diabetes Care (2016) 39(10):1830–45. doi: 10.2337/dc15-1182 27660122

[75] Fukazawa K Lee HT . Updates on hepato-renal syndrome. J Anesth Clin Res (2013) 4(9):352. doi: 10.4172/2155-6148.1000352 24459604 PMC3897161

