# Supplementary material for: Integrated analysis of potential gene crosstalk between non-alcoholic fatty liver disease and diabetic nephropathy
Source: Front Endocrinol (Lausanne). 2022 Oct 25;13:1032814. doi: 10.3389/fendo.2022.1032814 (PMC9642911; doi:10.3389/fendo.2022.1032814)

| **DN** | **RF predict NAFLD** | **RF predict non-NAFLD** |
| --- | --- | --- |
| **Diabetic nephropathy** | **44** | **18** |
| **Healthy controls** | **9** | **4** |

**Supplementary table 1: The prediction of RF model in DN**

**Supplementary table 2: Screened variables using LASSO Regression**

| **Genes** | **variation coefficient** |
| --- | --- |
| LPL | 0.21308371 |
| CD36 | 1.25441281 |
| F5 | -0.81974224 |
| SERPINF1 | 0.82045882 |
| C1orf21 | -1.02395206 |
| LAMP3 | 0.47848469 |
| AASS | 0.04177994 |
| RBBP4 | 0.35700811 |
| SPP1 | 0.05867598 |
| IL32 | 0.07585386 |
| GUSBP11 | 0.53261470 |
| FCN1 | 0.38926981 |
| CDC42EP2 | -1.49006417 |
| SLC35D2 | -1.81395272 |
| NFATC3 | -0.39350548 |
| CP | -1.51035314 |
| WIPI1 | 0.59357000 |
| RPS6KA1 | -0.71038958 |
| CBX7 | -0.36781714 |
| NEFM | -0.25013525 |
| TIMM17A | 0.94652035 |
| PTPRD | -0.26995763 |
| SLC26A4 | -0.21730360 |
| UQCRQ | 0.90186013 |
| S100A4 | 0.78328094 |
| ZDHHC3 | 1.03088238 |
| DPP6 | -1.63514465 |
| B2M | 0.15558907 |
| IFNW1 | -1.43813301 |
| MYO7A | 1.49037502 |
| MTCL1 | 0.25351667 |
| TPMT | -0.92263712 |
| PTPN3 | -0.35440667 |
| PSAP | 1.51175474 |

**Supplementary Figures:**

**Supplementary Figure1:**


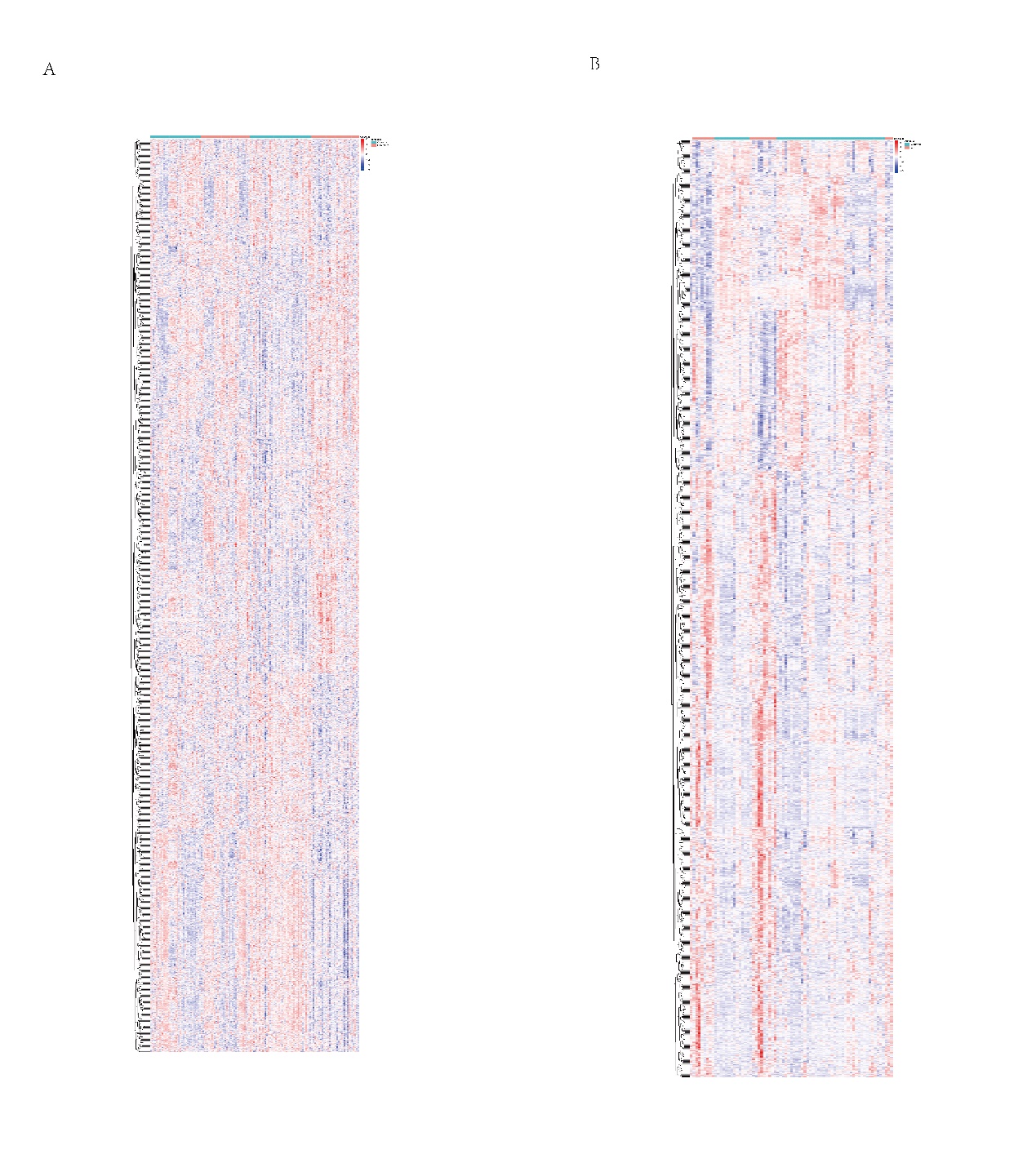


**Supplementary Figure2:**


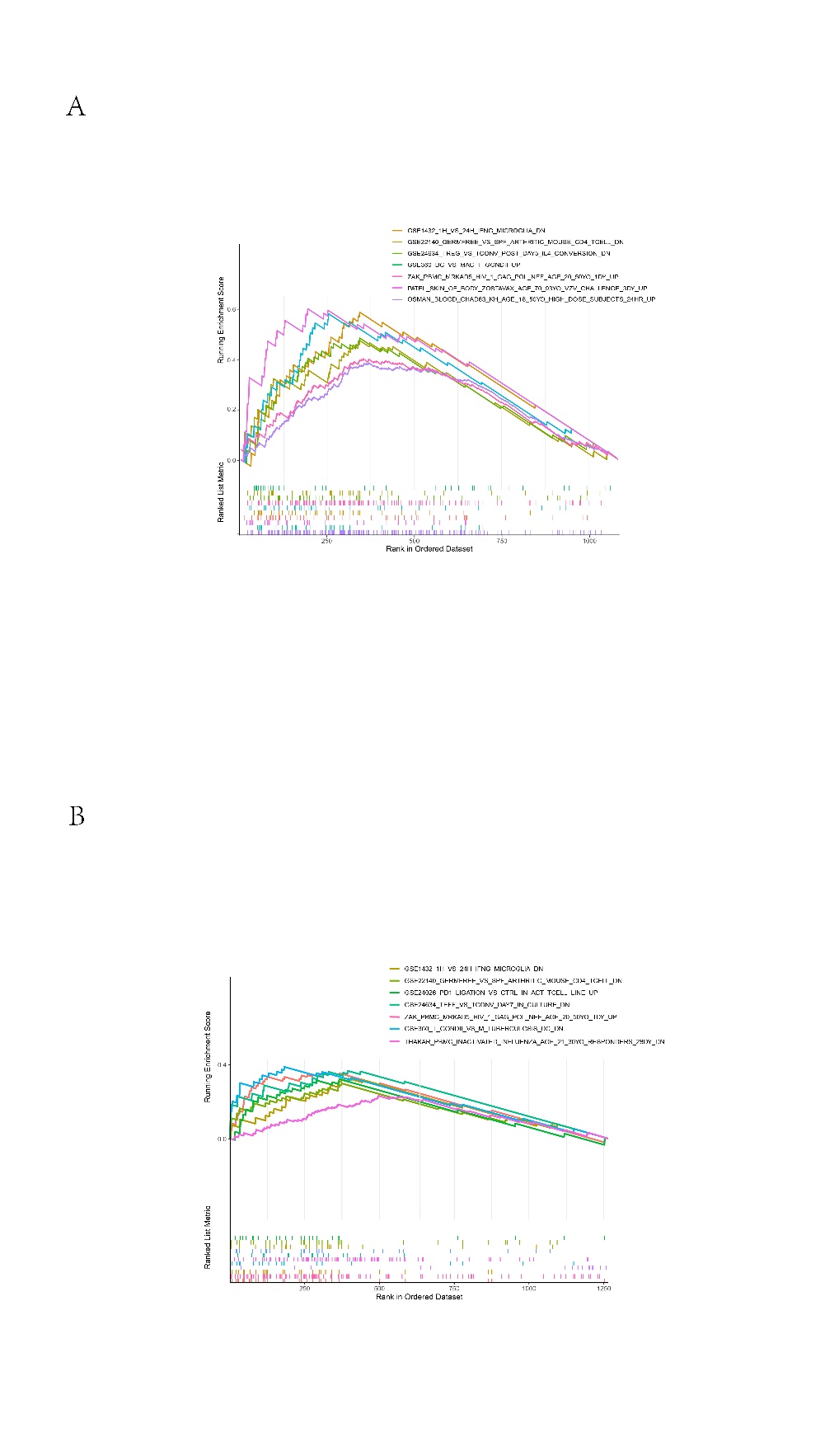


**Supplementary Figure3:**


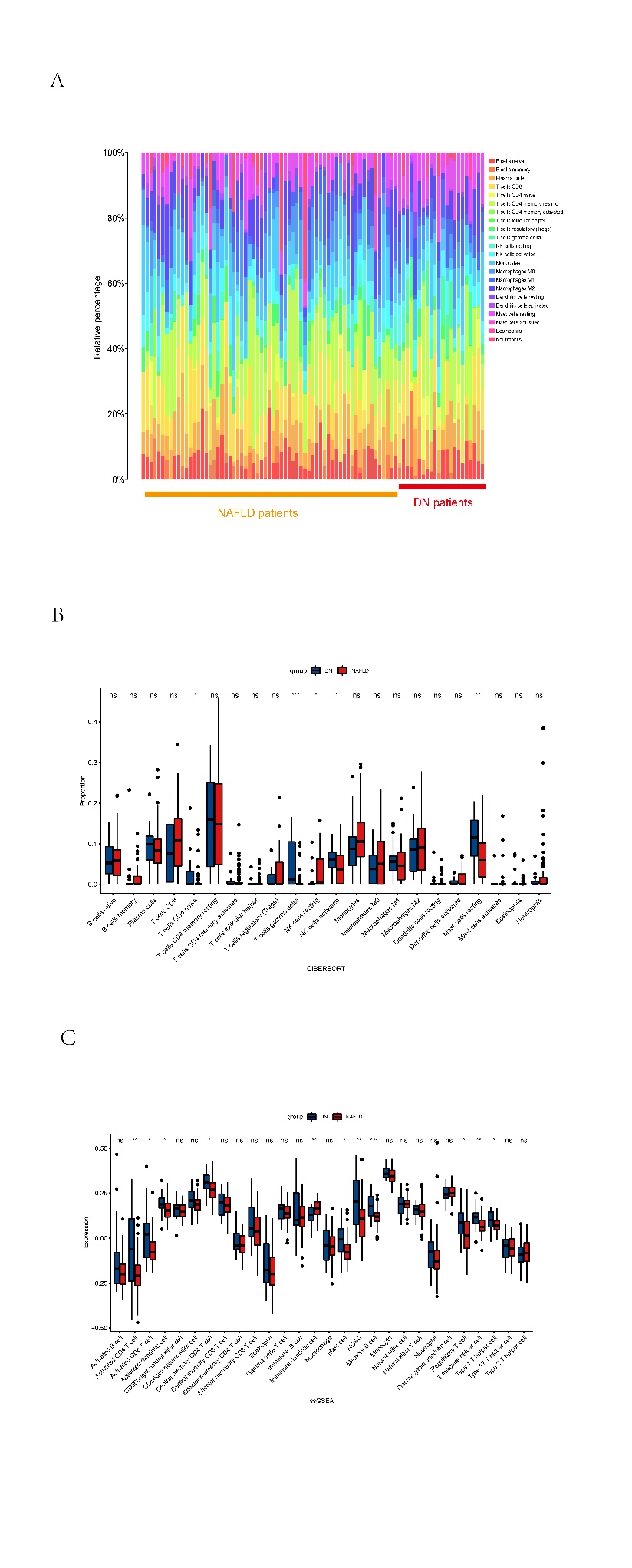


**Supplementary Figure4:**


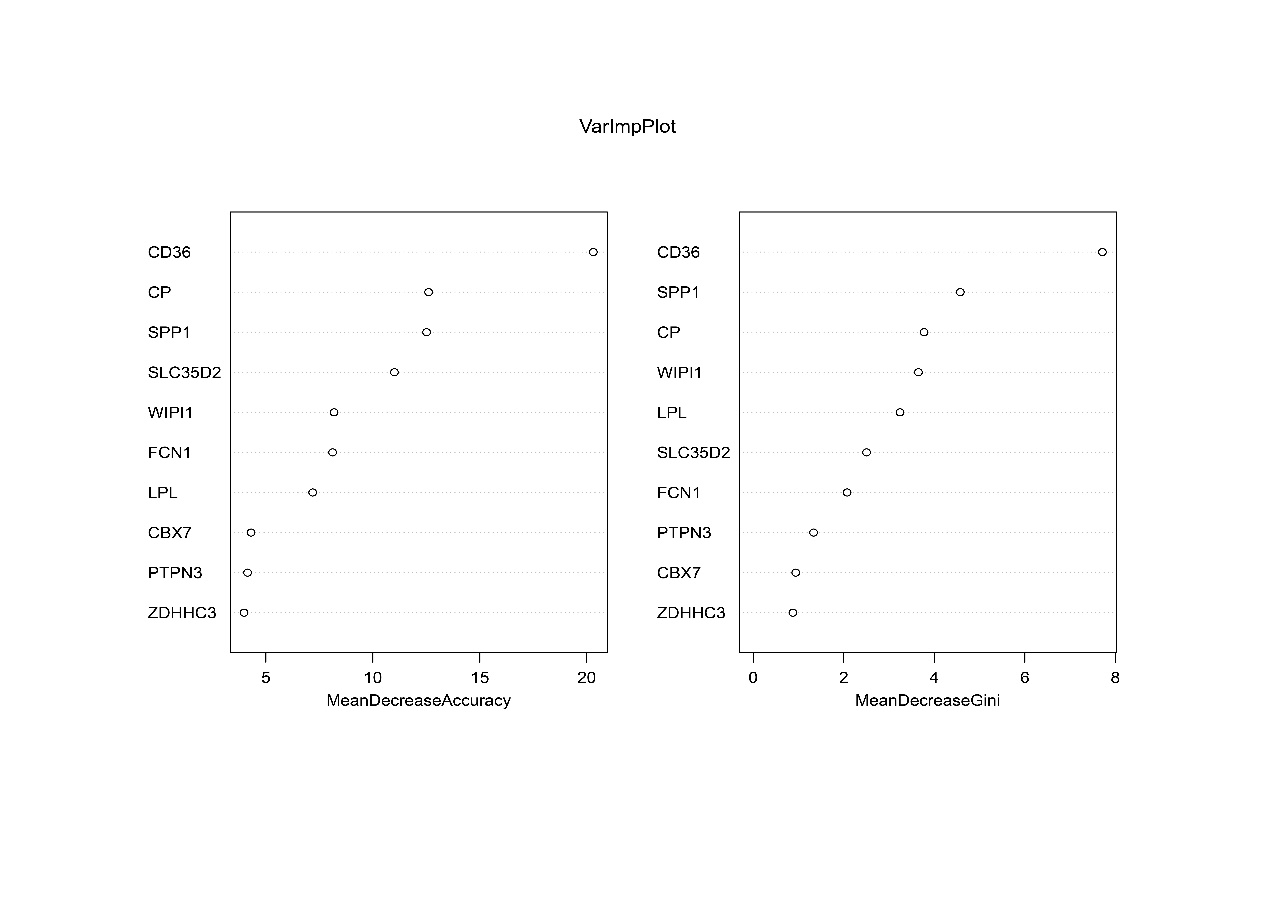


**Supplementary Figure5:**


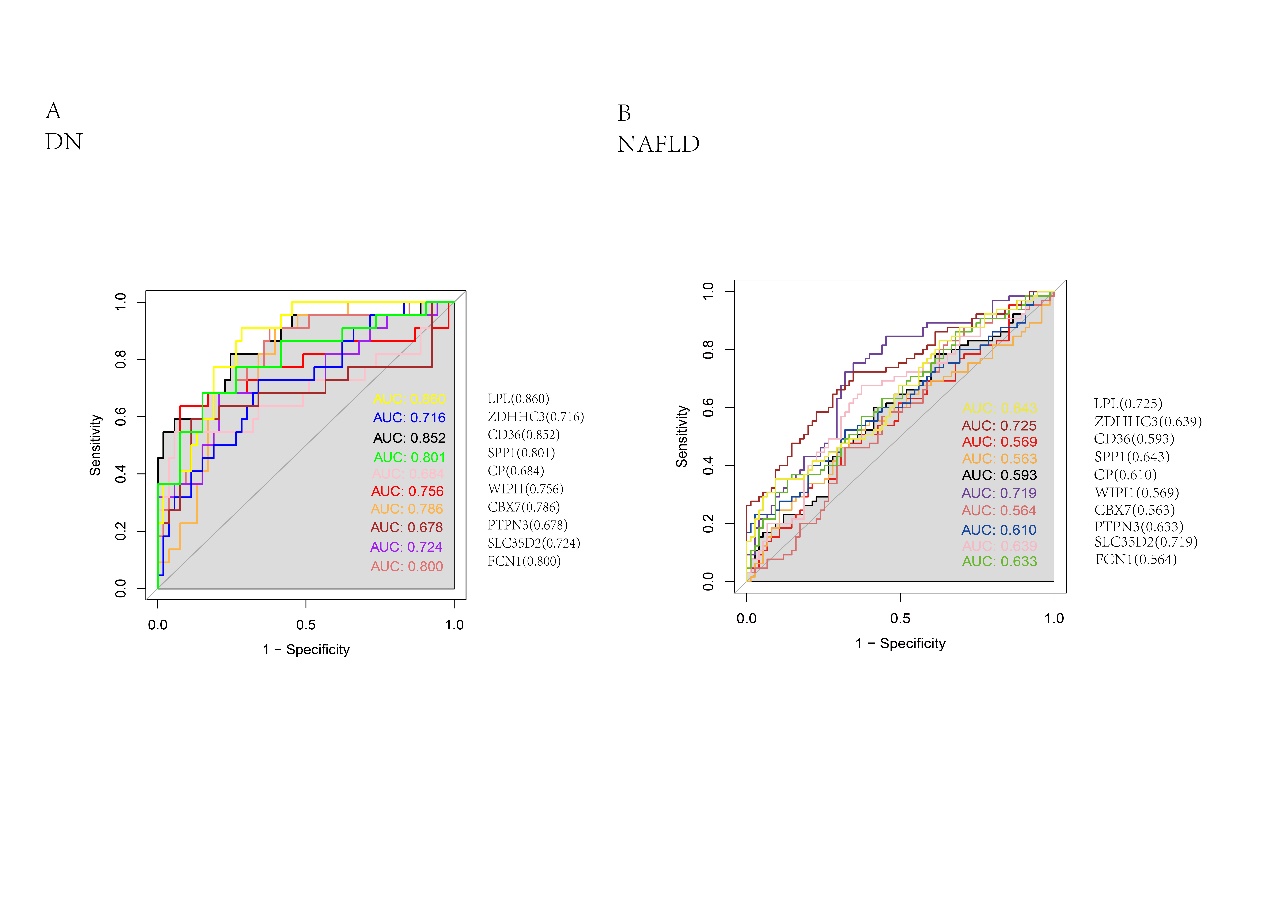


**Supplementary Figure6:**


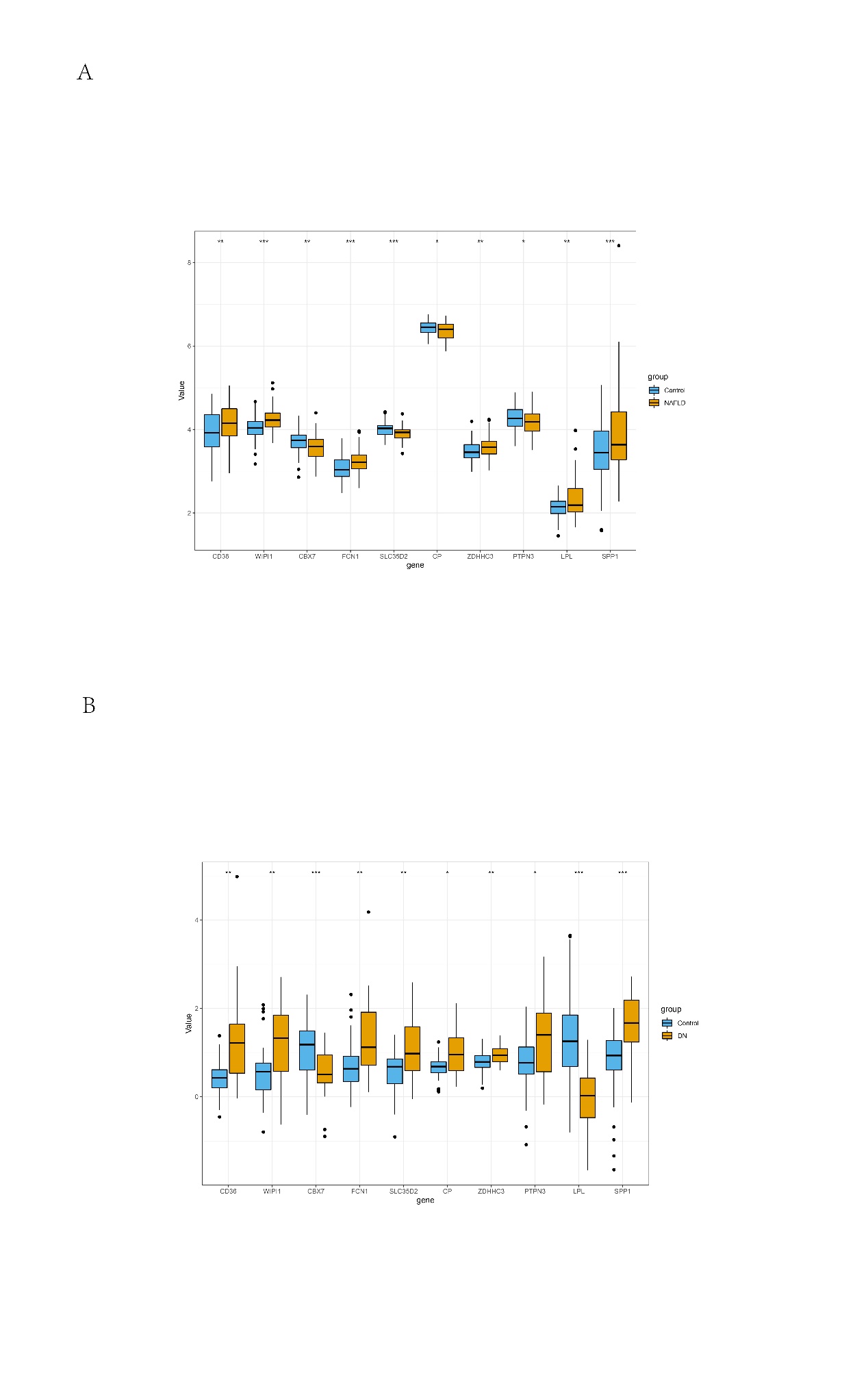


**Supplementary Figure7:**


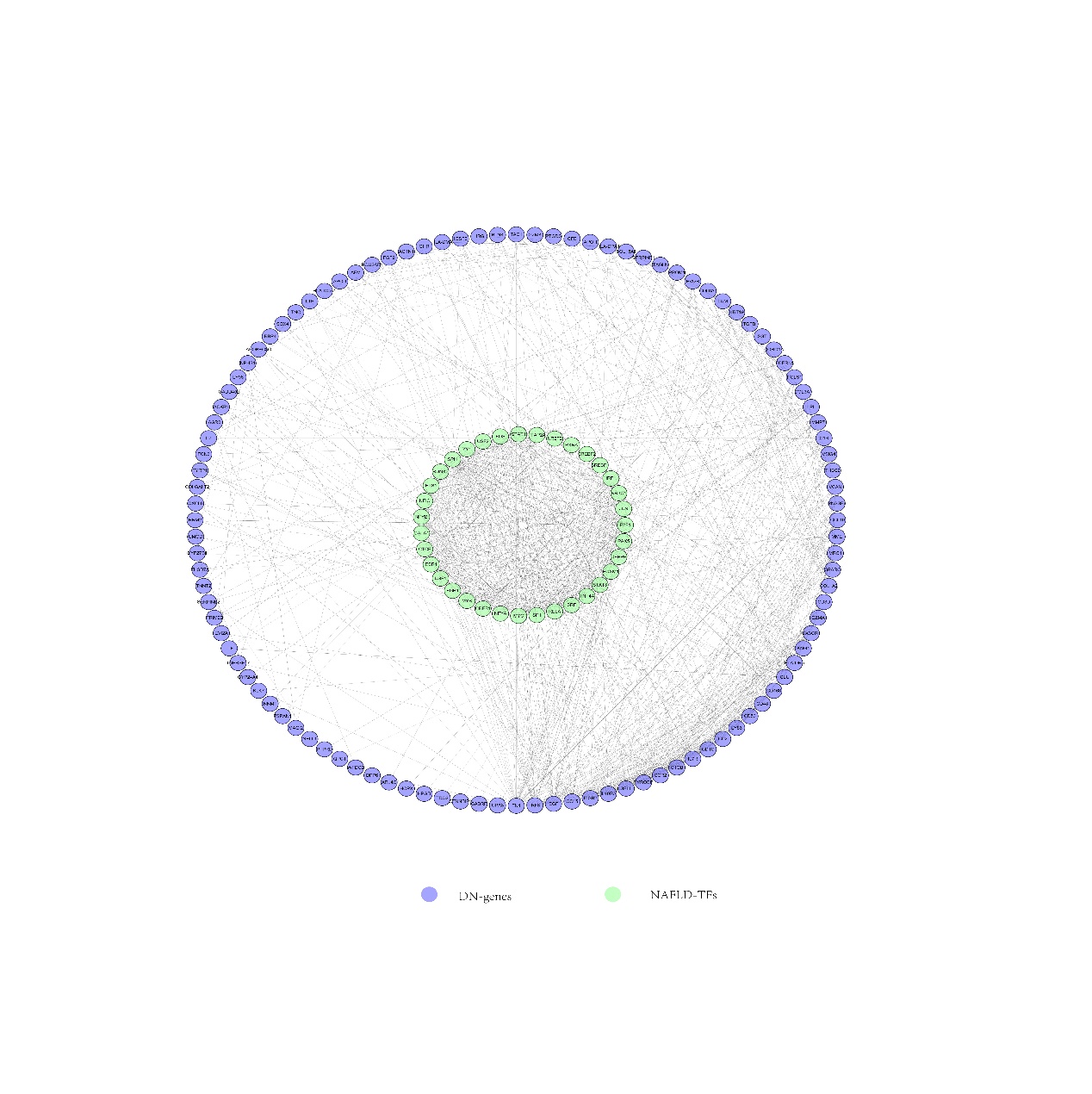

Supplement: Supplementary Figure 1 — The functional similarity between NAFLD and DN. (A) Comparison of functional enrichment of DN and healthy controls. (B) Comparison of functional enrichment of NAFLD and healthy controls. [file DataSheet_1.docx]
